# Fatigue and physical activity levels in poles living in Poland and the United Kingdom in the further year of the COVID-19 pandemic: a pilot study

**DOI:** 10.1186/s12889-023-17191-5

**Published:** 2023-11-16

**Authors:** Anna Zalewska, Monika Gałczyk, Aneta Mierzejewska

**Affiliations:** 1 Faculty of Health Sciences, University of Lomza, 14 Akademicka St, Lomza, 18-400 Poland; 2 Department of Psychology, Apsley Business School London, 2 Apsley House, 176 Upper Richmond Road, SW15 2SH London, UK

**Keywords:** Public health, Sport, Fatigue disorder, Emigration

## Abstract

**Objectives:**

The aim of this study was to conduct a preliminary assessment of the prevalence of fatigue and level of physical activity, as well as the relationship between fatigue and physical activity among Poles living in Poland and the United Kingdom (UK) in the further year in the COVID-19 pandemic.

**Methods:**

A web-based online survey was conducted among Poles living permanently in Poland and the UK in January 2023. Responses were obtained from 200 respondents aged 18–69 years. The level of fatigue was assessed by the Modified Fatigue Impact Scale (MFIS). The level of physical activity was measured by the International Physical Activity Questionnaire (IPAQ).

**Results:**

The median for the total fatigue score was equal to 17 points out of a possible 105. 13.5% of the subjects had a low level of physical activity, the average MET for high-intensity activity was 1294.55 METs, for moderate activity 714.44 METs, and for walking 631 METs. As age and number of COVID-19 cases increased, scores on the MFIS questionnaire scale also increased. With an increase in the number of COVID-19 cases, high-intensity MET scores decreased.

**Conclusions:**

The level of fatigue, in contrast to the level of physical activity, was low in the study population. There were few statistically significant differences in fatigue between people living in Poland and the UK. Further experimental studies on the physiological mechanism of differences in fatigue and physical activity are needed.

## Introduction

COVID-19, a viral infectious disease caused by the Wuhan coronavirus, has been known to the public for three years (SARS-CoV-2). It has affected over 450 million people worldwide, according to the World Health Organization [1].

Most SARS-CoV-2 virus patients recover completely within a few weeks. However, for some patients who have survived COVID-19, overcoming some of the symptoms is only the first step toward full recovery. Many studies in the literature [2–8] describe many post-COVID-19 symptoms, such as a loss of smell, visual disturbances, memory loss, and gastrointestinal problems. However, dyspnea and fatigue are the most common, and they frequently interfere with daily functioning [9–12].

Articles describing such phenomena under the term post-COVID are increasingly appearing in the literature due to certain physical or psychological symptoms reported by patients (fatigue, shortness of breath, cough, memory impairment, poor concentration) that persist for more than 12 weeks after infection. Post-COVID syndrome is defined by the National Institute for Health and Care Excellence (NICE) as “signs and symptoms developed during or after a Covid-19 compliant infection that last longer than 12 weeks and are not explained by a diagnosis” [13–15].

Although fatigue cannot be considered a discrete, single disease, the concept itself is a common phenomenon understood as a normal response to prolonged activity from which the body can recover on its own and which does not interfere with the performance of activities of daily living. However, symptoms of fatigue are usually more pronounced in people with certain illnesses or physical limitations than in healthy people [16–18].

The World Health Organization has revised its recommendations for physical activity for 2020.The most recent recommendations are 75 to 150 min of vigorous-intensity aerobic exercise or at least 150 to 300 min of moderate-intensity exercise per week for persons ages 18 to 64. WHO recommends people of all ages to lead less sedentary lives, which can have detrimental effects on humanity’s health and place a significant financial burden on society [19]. Lower levels of physical activity due to sedentary lifestyle, as well as a number of COVID-19 epidemic-related restrictions, such as the implementation of work or distance learning, mobility restrictions, or activity bans, may be associated with an increased risk of chronic cardiorespiratory, immunologic, and neuromuscular disease, as well as an increased risk of subjective fatigue, particularly in adults [20–24].

Patients recovering from COVID-19 disease frequently experience fatigue, which is defined as a decrease in physical and/or mental performance caused by changes in psychological, central, and peripheral factors caused by COVID-19 disease [25].

More study is required, and various forms of treatment are crucial, taking into account the interdependence between physical activity and exhaustion, in order to overcome this illness, which has a detrimental effect on the health of society worldwide [26, 27].

Notwithstanding Brexit and the COVID-19 pandemic, the Polish community in the UK still holds one of the top spots [28]. The authors of the study therefore decided to compare population samples from both countries.

According to research, migration among Poles living in the UK is not a source of dysfunctional state, nor does it cause identity problems or frustration [29]. Interesting observations conducted on migrants during the pandemic COVID-19 revealed that they had difficulties in accessing primary health care for various reasons [30]. At the same time, studies conducted among Poles living in the United Kingdom, who used health services in both their country of origin and their country of emigration during the pandemic, clearly showed that Polish health services were more attractive, both in terms of primary and specialized health care [31]. At the beginning of the pandemic, it was also observed that the feeling of threat related to the virus COVID-19 was stronger in distant areas (Europe and the world) than in Poland [32]. As it turned out, there was an increase in life satisfaction among a group of Polish migrants in the Netherlands during the pandemic COVID-19 [33]. The prevalence of fatigue may be different in various regions, and it may also be correlated with factors like age, gender, or exposure to chronic illnesses that may lead to exhaustion. This association has gained popularity, particularly in recent years when COVID-19 has emerged globally as one of the major contributors to population fatigue. The authors made the decision to look into the level of exhaustion in a sample of the Polish population that lived in remote areas. The authors did not find any studies of fatigue among samples of the same nation living in different geographical regions that were comparable to the one they used for this investigation. Thus, according to the authors, the study closes a gap and paves the way for generalizing to other groups and conducting an international investigation.

The aim of this study was to conduct a preliminary assessment of the prevalence of fatigue and level of physical activity, as well as the relationship between fatigue and physical activity among Poles living in Poland and the United Kingdom in the further year in the COVID-19 pandemic.

The following research questions, which are also particular objectives, were the primary focus of the investigation. Is there any correlation among the level of fatigue and age, gender, and level of physical activity, frequency of COVID and place of residence among Poles living in Poland and the United Kingdom?

## Materials and methods

### Participants and procedure

From 2 January to 31 January 2023, researchers conducted a pilot online survey of Poles living permanently in Poland and the United Kingdom. The survey in Polish was distributed in the form of a link to a Google form on the researchers’ social media accounts (the authors posted a survey on their personal social media profiles asking people to complete it) and Facebook groups for Poles living in the United Kingdom. The link provided details on the survey, participant anonymity, and informed consent. The participant had the right to withdraw at any moment. When the “Submit” box at the bottom of the form was clicked, it equated to giving informed consent to take part in the study. Only then was data entered. Responses were collected via Google forms. Inclusion criteria were: age over 18 years, consent to participate in the study, Polish citizenship, residence in Poland or the United Kingdom, Polish citizen with or without COVID-19, had received COVID-19 vaccine and a completed questionnaire. Exclusion criteria was suffering from depression, suffering from diseases of the nervous, musculoskeletal and respiratory systems. Responses were obtained from 200 respondents (104 from Poland and 96 from the UK) aged 18–69 years.

In sample calculation, to examine the prevalence of the low activity in each country, a formula was used to calculate the number of individuals to be included. If the number of individuals in the population is unknown, the formula n = (t^2^ x (pq)/d^2^) is used. For the calculations, a confidence interval of 0.95%, margin of error of 8% and 20% unknown prevalence was used (n = (t^2^ x (pq)/d^2^); p = prevalence of the event (probability) (0.5); q = prevalence of the absence of the event (1 - p) (0.5); t = theoretical value found on the t table at a certain degree of freedom and determined error rate (1.96); d = margin of error to be achieved based on the prevalence of the event (8% deviation, as 0.08)) [34]. With these assumptions, the sample size from each population - Polish and British - should be 97 people. The amount of collected data almost exactly meets these requirements.

University of Lomza Senate Committee on Ethics in Scientific Research gave its approval to the initiative (5,293,400). In accordance with Regulation (EU) 2016/679 of the European Parliament and of the Council of April 27, 2016, on the protection of natural persons with regard to the processing of personal data, on the free movement of such data, and repealing Directive 95/46/EC, in the Personal Data Protection Act of May 10, 2018, participation in the study was voluntary, and the findings were published (Journal of Laws 2018, item 1000). GDPR, or General Data Protection Regulation. The study’s goals, the poll’s methodology, and the relevant data protection rules were explained to the respondents.

### Methods of assessing the level of fatigue and physical activity

The research method was a diagnostic survey with metric and standardized instruments in the form of questionnaires: the Modified Fatigue Impact Scale (MFIS) and the International Physical Activity Questionnaire (IPAQ). Comprehensive statistical analyses of the findings from a survey of 200 respondents who resided in Poland and the United Kingdom were carried out in order to meet the study’s objective.

#### Modified fatigue impact scale

The Polish version of the Modified Fatigue Impact Scale (MFIS) was used to assess the level of fatigue [35]. The scale consists of three parts with questions related to the last four weeks. There are nine questions in Part F-1 that investigate the patient’s subjective feeling of how exhaustion affects their physical performance. Ten questions regarding the impact of fatigue on cognitive performance can be found in section F-2. Two questions on psychosocial function are found in F-3. The F-1 part can be scored from 9 to 45 points, the F-2 part from 10 to 50 points, and the F-3 part from 2 to 10 points, for a total score of 21 to 105 points. The higher the scores the respondent receives on the scale, the greater the impact of fatigue on his or her functioning. The value for Cronbach’s alpha reported in the publications is > 0.7 [36].

#### International physical activity questionnaire

The researchers assessed the level of physical activity using a shortened version of the International Physical Activity Questionnaire (IPAQ) in Polish [37]. It consists of 7 questions and is designed to survey individuals aged 15 to 69 years. Respondents give answers about daily physical activity at work, at home, in the environment, and in leisure time that lasts continuously for at least 10 min. Each activity’s description is given in MET-min/week units, which are calculated by multiplying the activity’s coefficient by the number of days it was conducted throughout the week and by the activity’s duration in minutes per day [27, 28]. Papers have reported a Cronbach’s alpha value of > 0.7 [38, 39].

### Statistical methods

The research assessed characteristics of a quantitative and qualitative nature. The analysis of each characteristic has its own specificity, namely the use of appropriate statistical tools for comparisons.

Basic descriptive statistics in the form of location measures and variability measures were calculated to characterize the variables studied. Spearman’s rank coefficients were calculated to determine the strength of correlations between variables. The significance of the differences was examined using a student’s t-test for independent samples. For variables measured on rank and nominal scales, the hypotheses that two qualitative characteristics are independent in the population were tested. This was accomplished using the chi-square test by Pearson. We not only examined if there is a link between the variables, but also how strong it is. Cramer’s V coefficients and Kendal’s tau b were applied as indicators of the relationship’s strength. For all analyses, a significance level of 0.05 was adopted as the baseline. All analyses were performed using the Statistica 13.1 package.

## Results

### General characteristics

The study concerns 200 Poles who are between the ages of 18 and 69 and reside in Poland (52%) and the United Kingdom (48%) (Table 1). Women barely outnumbered men in the study sample (51.5%). 90% of those surveyed had at least one case of COVID-19 from the pandemic, and 61.5% had received the SARS-CoV-2 vaccine.

**Table 1:** Characteristics of the study population

Feature	Class	Number of observations	% observations
**Age of respondents**	18–25 years	22	11.00
	26–35	55	27.50
	36–45	54	27.00
	46–55	48	24.00
	56–65	20	10.00
	over 66 years	1	0.50
**Gender**	woman	103	51.50
	man	97	48.50
**Country of residence**	Poland	104	52.00
	United Kingdom	96	48.00
**Number of incidences of COVID-19**	0	20	10.00
	1	84	42.00
	2	91	45.50
	3	5	2.50
**Vaccination against COVID-19**	not	77	38.50
	yes	123	61.50

### Fatigue level

According to analysis of the results in the table (Table 2) the median (mean) score on the scale assessing the effect of weariness on physical performance was fairly low, at 7 out of a possible 45 points. The median score on the scale used to assess how exhaustion affects cognitive skills was 9 out of a potential 50 points, which is a quite low level. For the results of the psychological scale, the median score was at a fairly low level equal to 2 points out of a possible 10. The median for the total score was equal to 17 points out of a possible 105.

**Table 2:** Basic descriptive statistics for the MFIS questionnaires

Variable	Average	Median	Minimum	Maximum	Std. dev.
**Physical functioning scale**	8.54	7.00	0.00	27.00	7.79
**Cognitive scale**	10.51	9.00	0.00	32.00	8.79
**Psychosocial scale**	1.85	2.00	0.00	8.00	1.89
**MFIS points**	20.90	17.00	00.00	63.00	18.05

MFIS - Modified Fatigue Impact Scale

### Level of physical activity

Each type of physical activity can be expressed in units of MET -min./week by multiplying the rate assigned to that activity by the number of days it is performed per week and the duration in minutes per day. Analysis of the results in the table (Table 3) showed that the average MET for high-intensity activity was 1294.55 METs, for moderate activity 714.44 METs, and for walking 631 METs.

**Table 3:** Basic descriptive statistics for the IPAQ questionnaire

Variable	Average	Median	Minimum	Maximum	Std. dev.
**MET high int**	1294.55	1200.00	400.00	2800.00	475.53
**MET moderate int**	714.44	600.00	200.00	1400.00	242.92
**MET walking**	631.34	528.00	66.00	2333.10	420.69
**Seating [min]**	343.75	300.00	60.00	840.00	152.13

MET - metabolic equivalent

After counting, the activity level of the subjects was determined with the use of a rank scale (Table 4). Analysis of the results in Table 4 revealed that 13.5% of the subjects had a low level of physical activity, 57% had a sufficient level and 29.5% had a high level.

**Table 4:** Level of physical activity

Level of physical activity	Number of observations (% of observations)
**high**	59 (29.5%)
**average**	114 (57%)
**low**	27 (13.5%)

### Correlations of fatigue levels and physical activity

In the following analyses, we checked whether the MFIS questionnaire results were related to the IPAQ questionnaire results. Analysis of the results in the table (Table 5) revealed that there are statistically significant negative correlations p < 0.05 for the scores of MET and IPAQ compared to the scores of the individual scales of the MFIS questionnaire. It can be concluded that as the scores of the MFIS questionnaire scales and its summary score increased (scores worsened), the MET scores and the level of physical activity decreased. In the case of sitting compared to the scores of the individual scales of the MFIS questionnaire, there were statistically significant positive correlations p < 0.05. It can therefore be concluded that as the scores for the scales and the sum score of the MFIS questionnaire worsened, the respondents spent more and more minutes sitting.

**Table 5:** Respondents Spearman rank order correlation of MFIS and IPAQ

Pair of variables	R Spearman	t (N-2)	p
**MET high int & scale physical functioning**	-0.22	-2.88	0.0044
**MET high int & Cognitive scale**	-0.20	-2.67	0.0084
**MET high int & Psychosocial scale**	-0.19	-2.52	0.0125
**MET high int & MFIS pkt**	-0.22	-2.88	0.0045
**MET moderate int & scale physical functioning**	-0.32	-4.56	0.0001
**MET moderate int & Cognitive scale**	-0.27	-3.69	0.0003
**MET moderate int & Psychosocial scale**	-0.35	-4.97	0.0001
**MET moderate int & MFIS pt**	-0.30	-4.27	00001
**MET walking & scale physical functioning**	-0.22	-3.05	0.0027
**MET walking & cognitive scale**	-0.29	-4.07	0.0001
**MET walking & psychosocial scale**	-0.10 -1.42 0.1572	-1.42 0.1572	0.1572
**MET walking & MFIS points**	-0.26 -3.60 0.0004	-3.60 0.0004	0.0004
**Sitting & Scale Physical Functioning**	0.23 3.20 0.0016	3.20 0.0016	00016
**Sitting & Cognitive Scale**	0.29 4.15 0.0000	4.15 0.0000	0.0001
**Sitting & Psychosocial Scale**	0.16 2.24 0.0262	2.24 0.0262	0.0262
**Seat & MFIS pt**	0.27 3.79 0.0002	3.79 0.0002	0.0002
**Physical Activity Level & Physical Functioning Scale**	-0.17	-2.48	0.0141
**Physical Activity Level & Cognitive Scale**	-0.17	-2.41	0.0167
**Physical activity level & Psychosocial scale**	-0.14	-2.04	0.0425
**Physical activity level & MFIS points**	-0.18	-2.51	0.0130

MET - metabolic equivalent, MFIS - Modified Fatigue Impact Scale

In the subsequent analyses, it was checked whether the age of the subjects was correlated with the results of the MFIS questionnaire. Analysis of the results in the table (Table 6) showed that age was significantly correlated with the MFIS questionnaire scores p < 0.05. It can be concluded that as the subjects’ age increased, the MFIS questionnaire scale scores increased, i.e. worsened. Two statistically significant correlations were also found between age and the scores for MET high intensity and MET medium intensity. This suggests that with increasing age, the scores for these characteristics decreased.

**Table 6:** Respondents Spearman rank order correlation of age, MFIS and IPAQ

Pair of variables	R Spearman	t (N-2)	p
**Physical functioning & age**	0.37	5.58	p < 0.0001***
**Cognitive scale & age**	0.34	5.06	p < 0.0001***
**Psychosocial scale & age**	0.39	6.05	p < 0.0001***
**MFIS pt & age**	0.36	5.43	p < 0.0001***
**MET high int & age**	-0.17	-2.24	0.0267*
**MET moderate int & age**	-0.24	-3.32	0.0011***
**MET walking & age**	0.04	0.60	0.5509
**Seating & age**	0.07	0.99	0.338
**Physical activity level & age**	-0.02	-0.27	0.7892

p - test probability value test, p < 0.05 (*), p < 0.001 (***), MFIS - Modified Fatigue Impact Scale, MET - metabolic equivalent

Subsequently, the number of incidences of COVID-19 in the subjects was related to the results of the MFIS and IPAQ questionnaires. Analysis of the results in the table (Table 7) showed that the number of incidences was positively statistically significantly correlated with the MFIS questionnaire results p < 0.05. It can be concluded that as the number of COVID-19 incidents increased among subjects, MFIS questionnaire scale scores increased, i.e., worsened. A statistically significant relationship was also found between the number of incidents and MET high intensity scores. It can be concluded that as the number of cases increased, MET high intensity scores decreased.

**Table 7:** Respondents Spearman rank order correlation of incidences of COVID-19, MFIS and IPAQ

Pair of variables	R Spearman	T (N-2)	p
**Physical functioning scale & number of incidences of COVID-19**	0.28	4.04	0.0001***
**Cognitive scale & number of incidences of COVID-19**	0.32	4.71	p < 0.0001***
**Psychosocial scale & number of incidences of COVID-19**	0.17	2.50	0.0133**
**MFIS pt & number of incidences of COVID-19**	0.29	4.22	p < 0.0001***
**MET high int & number of incidences of COVID-19**	-0.16	-2.13	0.0345*
**MET moderate int & number of incidences of COVID-19**	-0.08	-1.13	0.2591
**MET walking & number of incidences of COVID-19**	-0.12	-1.67	0.0972
**Sitting & number of incidences of COVID-19**	0.19	2.62	0.0095**
**Physical activity level & number of incidences of COVID-19**	-0.11	-1.49	0.1386

p - test probability value test, p < 0.05 (*), p < 0.01 (**), p < 0.001 (***), MFIS - Modified Fatigue Impact Scale, MET - metabolic equivalent

It was also verified whether gender significantly differentiated the MFIS and IPAQ questionnaire scores. Analysis of the results in the table (Table 8) found statistically significant differences only in the variable MET moderate intensity p < 0.05. Women were found to have statistically significantly lower mean MET moderate intensity scores.

**Table 8:** Student’s t-test for independent samples gender, MFIS and IPAQ

Variable	Mean ± SD female	Mean ± SD male	t	df	p
**Physical functioning scale**	8.50 ± 7.44	8.59 ± 8.19	-0.08	198	0.93
**Cognitive scale**	10.37 ± 8.12	10.66 ± 9.49	-0.23	198	0.82
**Psychosocial scale**	1.83 ± 1.84	1.87 ± 1.94	-0.12	198	0.91
**MFIS points**	20.70 ± 16.90	21.11 ± 19.28	-0.16	198	0.87
**MET high int**	1239.51 ± 484.17	1347.62 ± 463.74	-1.47	163	0.14
**MET moderate int**	676.92 ± 243.62	752.81 ± 237.45	-2.12	178	0.036*
**MET walking**	629.33 ± 464.89	633.39 ± 372.69	-0.07	186	0.95
**Seat**	329.38 ± 144.63	358.13 ± 158.73	-1.31	190	0.19

p - test probability value test, p < 0.05 (*), MFIS - Modified Fatigue Impact Scale, MET - metabolic equivalent

Subsequent verification analyses examined whether the country of residence made a significant difference in MFIS and IPAQ questionnaire scores (Table 9). Analysis of the results revealed that individuals residing in Poland had statistically significantly higher (i.e., worse) mean scores for the cognitive scale and minutes of sitting than individuals residing in the United Kingdom.

**Table 9:** Student’s t-test for independent samples country of residence and MFIS and IPAQ

Variable	Mean ± SD Poland	Mean ± SD UK	t	df	p
**Physical functioning scale**	9.26 ± 7.45	7.76 ± 8.11	1.36	198	0.17
**Cognitive scale**	11.89 ± 8.38	9.01 ± 9.02	2.34	198	0.020*
**Psychosocial scale**	2.00 ± 1.90	1.69 ± 1.87	1.17	198	0.24
**MFIS points**	23.15 ± 17.05	18.46 ± 18.86	1.85	198	0.07
**MET high int**	1286.08 ± 575.29	1302.33 ± 363.96	-0.22	163	0.83
**MET moderate int**	746.24 ± 296.56	680.46 ± 162.71	1.83	178	0.07
**MET walking**	657.54 ± 534.32	602.80 ± 243.14	0.89	186	0.37
**Seat**	392.35 ± 170.89	288.67 ± 103.69	5.00	190	p < 0.0001***

p - test probability value test, p < 0.05 (*), p < 0.001 (***), MFIS - Modified Fatigue Impact Scale, MET - metabolic equivalent

It was also examined whether the country of residence was associated with physical activity levels. For this purpose, Pearson’s χ2 independence tests were used to test the association. The analysis of the results in the table (Table 10) gave no reason to reject the null hypothesis of independence of the variables. It can be concluded that the respondents’ country of residence is not statistically significantly associated with activity level p > 0.05.

**Table 10:** Summary bivariate table: observed frequencies

Level of physical activity	Country of residence		Row total
	Poland	UK	
**low**	19	8	27
**% columns**	18.27%	8.33%	
**sufficient**	55	59	114
**% columns**	52.88%	61.46%	
**high**	30	29	59
**% columns**	28.85%	30.21%	
**General**	104	96	200

χ2 = 4.45; df = 2; p = 0.11

## Discussion

Fatigue is a problem that increasingly affects younger people and has a number of negative consequences [40, 41]. During the most difficult period of the COVID-19 pandemic and the global lockdown, it was considered one of the most commonly reported consequences of the pandemic [42, 43]. The problem remains poorly understood, and there is no consensus on its definition or a clear causal mechanism [44]. In the authors’ own study, the overall severity of fatigue according to the Modified Fatigue Impact Scale (MFIS) was 17, suggesting a low level. In studies conducted directly during the pandemic COVID-19 or in patients diagnosed with chronic COVID-19, the level of estimated fatigue was at a much higher level [43, 45, 46]. It is difficult to comment on these results because no studies were found in the literature that were consistent with the authors’ study.

Regular physical activity and an adequate diet should be essential for maintaining good health [47]. During lockdown, it was also found that this may also lead to reduced fatigue associated with pandemic constraints [48]. Regular physical activity is associated with better physical and mental health and better stress management [49]. In our study, we found that the level of physical activity decreased as the MFIS questionnaire scale increased. A study conducted on Polish students showed the adverse health effects of limited physical activity in all areas considered. Respondents who did not abstain from physical activity were less prone to fatigue than those who reduced their physical activity even minimally [43]. Similar associations are also found in the results of studies conducted in different parts of the world [25, 50, 51]. Regular physical activity has also been indicated as a factor that may play a protective role against fatigue [43]. In our pilot study, only 13.5% of respondents reported low levels of physical activity, 57% had adequate levels, and 29.5% had high levels. The country of residence had no effect on the overall level of physical activity of the Poles surveyed. Physical activity during the COVID-19 pandemic was identified as one of the main factors in the impact of fatigue on general and physical health. Observational studies have shown that fatigue (among other factors) leads to lower levels of physical activity [52]. However, the results of this study could not show a causal relationship, since physical activity can influence fatigue and vice versa.

It also shows that as the MFIS scale worsened, respondents spent more and more minutes sitting. Several previous studies, even before the pandemic outbreak of the COVID-19, have shown the adverse effects on fatigue levels of too much time spent sitting [53–55].

Our study also found two statistically significant associations between age and MET scores for high and moderate intensity. As age increased the scores for these traits decreased. These observations are consistent with a systematic review of physical activity, sedentary behaviour, and exercise in adults by gender and age [56].

The severity of fatigue was inversely related to age, which has been confirmed in other studies [25]. Other results were obtained in Egypt, where no significant association was found between fatigue and age in COVID-19 subjects. Overall, the association of fatigue with age is inconsistent among available subjects [45, 57, 58].

As the number of COVID-19 cases in the subjects increased, the MFIS questionnaire scale scores worsened, which is consistent with other studies conducted in Sweden [59].

In our study, gender differences in physical activity behaviour were found among the respondents. The gender of the respondents was found to have an influence on the scores of MET. It was found that women had statistically significantly lower average MET moderate intensity scores. This is consistent with other studies where women reported high levels of low physical activity compared to men [51].

Gender had no influence on MFIS scores in our study. It seems that there are conflicting results in the literature where female gender [45, 51] or male gender is identified as an important predictor of fatigue [60] and also studies where gender has no influence on fatigue scores [46]. The authors agree that lower levels of fatigue are associated with significantly higher levels of personal resilience and coping skills, which characterize the male gender [61].

The study presented here has several limitations that should be noted. The first is the cross-sectional nature of the study, which cannot provide causal evidence for the observed relationships. The second limitation is the lack of data on levels of fatigue and physical activity before and at the outbreak of the pandemic, which might be relevant to the levels of the variables analyzed. Another limitation was the use of subjective methods to measure fatigue and physical activity. The authors are also aware that a limitation was the small sample size and the conduct of the study as an online survey, which may have limited the generalizability of the results. Despite these limitations, the study presented here also has a number of strengths, such as increasing knowledge about fatigue and physical activity in the second year of the pandemic among Poles living in Poland and the UK. The use of standard and validated instruments, easy access to the study group, and low costs were also strengths of the present study.

## Conclusions

The pilot study showed that there were few statistically significant differences in fatigue between people living in Poland and the UK. The level of fatigue in the study population was low. With increasing age and number of COVID-19 cases, MFIS scale scores increased, i.e., worsened. At the same time, as the number of COVID-19 cases increased, the high-intensity MET scores decreased. Women were found to have statistically significantly lower mean MET moderate intensity scores. It was found that individuals residing in Poland had statistically significantly higher (i.e., worse) mean scores for the cognitive scale and minutes of sitting than individuals residing in the United Kingdom as well as the respondents’ country of residence is not statistically significantly associated with physical activity level p > 0.05.

Further experimental studies on the physiological mechanism of differences in fatigue and physical activity are needed. In relation to the findings of our own research, physical activity interventions are recommended to address the unequal distribution of fatigue prevalence between people with high and low levels of physical activity.

## Data Availability

The data that support the findings of this study are openly available in RepOD, 10.18150/0DR19C (accessed on 20 February 2023).

## Abbreviations

IPAQ: International Physical Activity Questionnaire
MFIS: Modified Fatigue Impact Scale
UK: United Kingdom
MET: Metabolic Equivalent
